# The large-scale expansion of rubber plantations in southern India: major impacts and the changing nature of drivers

**DOI:** 10.1007/s10661-024-12517-1

**Published:** 2024-03-11

**Authors:** Dhanya Vijayan, Renoy Girindran, Anu Susan Sam, Archana Raghavan Sathyan, Harald Kaechele

**Affiliations:** 1 Leibniz Centre for Agricultural Landscape Research (ZALF), Eberswalder Str. 84, 15374 Müncheberg, Germany; 2 School of Geography, University of Nottingham, Nottingham, NG7 2 UK; 3 Kerala Agricultural University, Regional Agricultural Research Station, Kumarakom, Kerala 686563 India; 4 College of Agriculture, Kerala Agricultural University, Vellayani, Kerala India; 5 Eberswalde University for Sustainable Development, Schicklerstrasse 5, 16225 Eberswalde, Germany

**Keywords:** Land use change, Environmental drivers, Changing nature of drivers, Data scarce region, Geospatial techniques

## Abstract

This study investigates the major environmental and socio-economic impacts of an increase in the area of rubber plantations and the changing patterns of drivers of land use changes. Using a combination of geospatial techniques and socio-economic methods, we mainly analyzed the rate of increase in area under rubber plantations, the major impacts of land use changes, and the changing drivers of land use changes. Our results show that the area under rubber plantations has increased significantly within the study area, with the area under rubber plantations increasing from 30 to 74% of the total area within five decades. Impact assessment of land use changes based on household surveys showed significant improvement in the socio-economic conditions of the farmers, however, at the expense of severe environmental degradation. Our results also indicate that while areas under rubber plantations continue to increase, the drivers of land use changes have changed over time. Furthermore, it has been observed that in the past, many interventions prioritized social and economic development and placed less emphasis on the ecological stability of the region. Perceptions of farmers revealed that the effects of ecological fragility already affected the economic robustness of the whole area. Therefore, we conclude that government interventions to support additional rubber cultivation should also focus on ecosystem stabilization in order to minimize the risk of an ecological catastrophe that would significantly affect the economic prosperity of the region.

## Introduction

Land use and land cover (LULC) changes are key focus areas in global environmental change research (Liu et al., 2016) and a significant manifestation of human activity having an impact on the natural environment (Zhao et al., 2018). Agricultural practices are considered as one of the major causes of human-induced land use changes and exert massive pressure on natural resources (Benini et al., 2010). Commercialization of agriculture has led to the expansion of area under cash crops such as rubber, oil palm, coffee, and cashew, replacing traditional food crops (Antje et al., 2015). In tropical rural areas, the production of cash crops often provides more profit to the farmers in comparison to subsistence crops (Sayer et al., 2012). Among the cash crops, rubber (*Hevea brasiliensis*) is the prominent player that replaced the traditional agricultural crops in the tropics. Attracted by the economic benefits and incentives resulting from the steady increase in worldwide consumption of rubber in the last decades, many farmers have switched the traditional farming areas to rubber cultivation, and nowadays, rubber plantations are rapidly expanding in both climatically optimal and sub-optimal environments in almost all rubber growing countries (Fox, 2014; Chattopadhyay, 2015).

Land use conversions in an area can affect the environmental stability of a region, and the main concerns about land use change are related to negative environmental, economic, and social impacts (Alijani et al., 2020; Vijayan et al., 2021; Zhao et al., 2022). One of the most important environmental impacts of the expansion of area under rubber plantations is the change in local and regional water regimes (Guardiola-claramonte et al., 2008 & 2010; Tan et al., 2011; Ma et al., 2019). For instance, studies show that rubber trees consume more water than the native species (Ayutthaya et al., 2011); they increase evapotranspiration (Tan et al., 2011), reduce surface water run-off and stream flow and water yield (Guardiola-Claramonte et al., 2008; Celine et al., 2015), cause drying up of wells (Qiu, 2009), and lead to land degradation and soil erosion (Liu et al., 2013; Wu et al., 2016). Studies have also shown that the increase in rubber plantations has socio-economic benefits, such as the increase in per capita income and expenditure, as well as overall household income among rural communities (Liu et al., 2006). However, this also leads to a reduction in plant diversity and loss of traditional agriculture (Häuser et al., 2015).

Land use change is the cause and effect of global environmental change, and it is driven by numerous factors, including biophysical and socio-economic factors (Song et al., 2018; Turner et al., 2007). Anthropogenic activities undoubtedly play a dominant role in changing landscapes, and about 60% of global land changes are linked to direct human activities (Song et al., 2018). Understanding the major impacts and key drivers of land use changes in a region is vital for specific planning and sustainable land management (Arowolo & Deng, 2018; Wang et al., 2022). Drivers of land use changes are complex and dynamic and vary from one location to another in relation to the socio-economic and biophysical factors of that place (Li et al., 2016). Furthermore, they are also time-specific and may change over time, and it is difficult to generalize these drivers (Beilin et al., 2014; Munthali et al., 2019). Therefore, understanding the dynamics of land use change and their drivers spatially and temporally is essential for reducing environmental and socio-economic challenges as well as for proper land use management (Foley et al., 2005).

India is one of the major victims of the developing world, facing unprecedented pressure due to LULC shifting to exotic crops such as rubber. India is the fourth largest producer of natural rubber after Thailand, Indonesia, and China. In India, the state of Kerala accounts for 92% of total rubber production and 84% of the area under rubber cultivation (Karunakaran, 2017). Kerala, a tropical state in South India, is an example of a region with a dynamic history of land use changes that has not been well documented (Fox et al., 2017). In Kerala, rapid agricultural land use changes occurred since the 1970s, and it coincides with the introduction of rubber plantations. Originally, rubber cultivation was introduced into areas with degraded forests; from there, it spread all over and replaced natural vegetation and other crops, viz. tapioca, cashew nut, fruit trees, and coconut (Chattopadhyay, 2015). Today, about 14% of Kerala’s total geographical area is occupied by rubber, which constitutes 21% of the total cropped area of the state. However, so far, no studies have attempted to understand the major impacts and drivers of the large-scale expansion of rubber plantations in rural Kerala.

The absence of relevant datasets is one of the major constraints in assessing the impacts of expansions of rubber plantations and their drivers in rural tropics including rural Kerala. Geospatial technologies can be used to monitor and assess historical land use conversions within an area. Whereas there is no data available for understanding major impacts and drivers. In such cases, socio-economic methods, especially understanding the perceptions of local communities, can be a good tool for understanding the dynamics of LULC changes. Although the application of socio-economic methods in the field of environmental and natural resource management is increasing, its potential to combine with other disciplines is not adequately addressed (Gunnell et al., 2017). Especially combining geospatial techniques with knowledge of local communities is capable to monitor the linkages between the LULC changes and the changes in environmental resources and ecosystem services (Malek & Boerboom, 2015; Zaehringer et al., 2018; Delgado-Aguilar et al., 2019).

The present article attempts to illustrate the intensive land use changes in Kerala, with special emphasis on the expansion of rubber plantations, by combining geospatial techniques and socio-economic methods. The major objective of this study is to provide important insights, impacts, and drivers of rubber plantation expansion.

## Materials and methods

### Study area

**Fig. 1:**
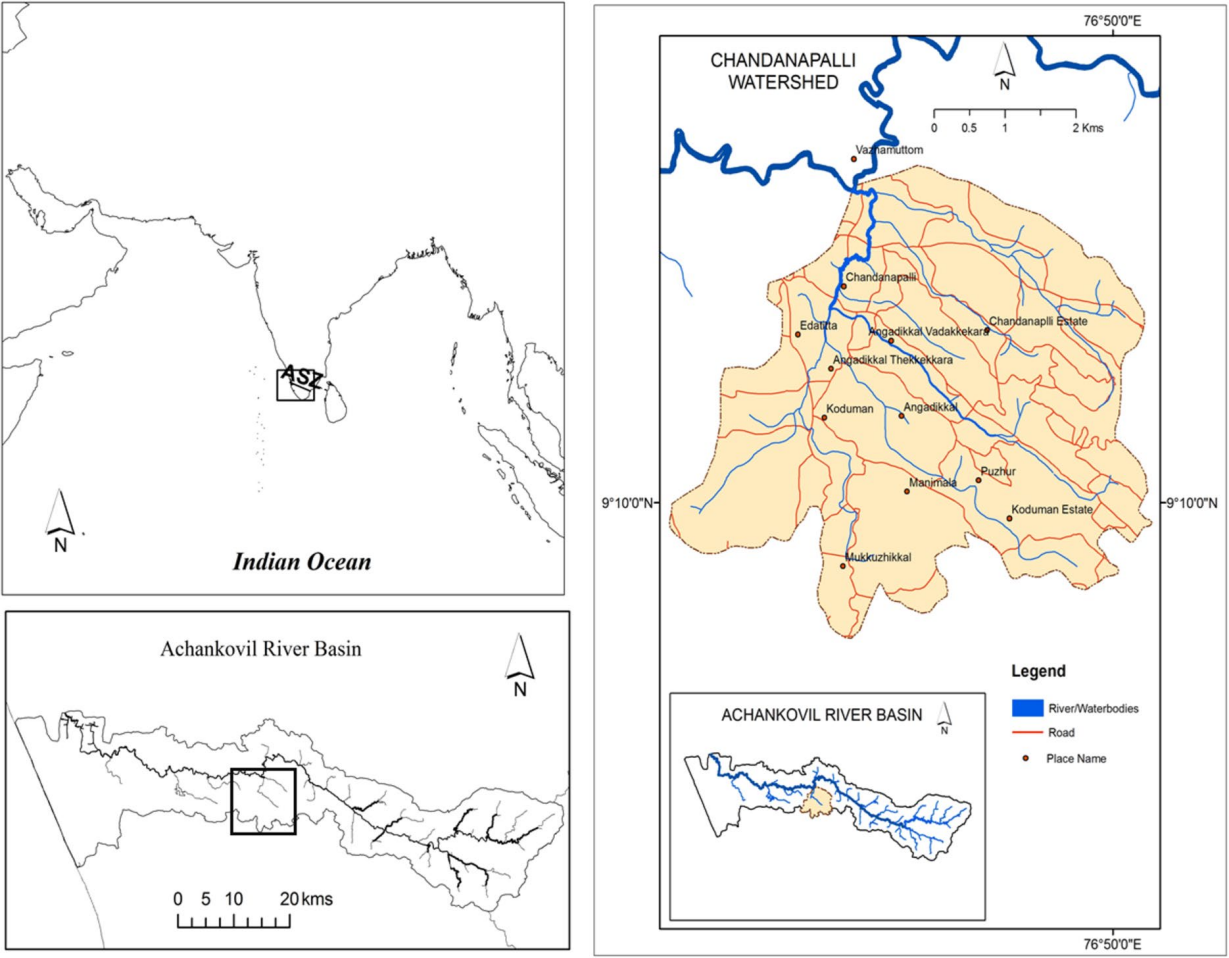
Location of Chandanapalli sub-watershed

The study area is the Chandanapalli sub-watershed, which is located in the midlands of Kerala, India (Fig. 1). It is the largest sub-watershed of the Achankovil river basin and has a total area of 66.24 km^2^. The elevation of the basin ranges from 20 to 200 m above mean sea level (Dhanya & Renoy, 2015). Rubber is the major plantation crop cultivated in this watershed. In 1962, the Plantation Corporation of Kerala Limited (PCKL), which is a public undertaking plantation company of the state government, had started rubber plantations in the Chandanapalli sub-watershed. PCKL started two rubber estates, namely Chandanapalli and Koduman, within the boundary of the watershed. These two estates together constitute an area of about 29 km^2^; out of which, 19.8 km^2^ fall within Chandanapalli sub-watershed.

Chandanapalli was selected as it undergoes a high rate of land use conversion to rubber plantation, and it represents sub-regional conditions of the entire midland area of Kerala. After 1967, individual households started planting rubber trees in their plots, and at present, about 80% of the watershed area is under rubber plantations. These land use changes may have altered the ecosystem services, resulting in a severe impact on the environment. The major concerns due to this land use conversion are a reduction in the cultivation of major food crops, soil degradation, water scarcity, drought in summer months, etc. Therefore, this sub-watershed is an ideal case for analyzing the key impacts of rubber plantation expansion and its drivers.

### Methodology

A combination of geospatial techniques, focus group discussions, household surveys, and analysis of farmers’ perceptions was used to achieve the objectives of the study. A summary of the research methods adopted is given below in Table 1.

**Table 1:** Summary of research methods

Components	Data source/participants	Purpose
Topographic maps and satellite data	Survey of India toposheets; LANDSAT images	To identify the extent of land use change and the rate of expansion of rubber plantation
Household survey	40 households (183 residents)	To identify major environmental and socio-economic impacts of land use changes within the study area
Expert interviews and focus group discussions	Five experts with in-depth knowledge of land use changes within the area and 3 focus groups (8–12 rubber farmers)	To understand the major drivers of the expansion of rubber plantations within the study area

#### Mapping of the area under rubber plantation and change assessments

Datasets used to map the expansion of area under rubber plantation within the sub-watershed included the following: (1) survey of India (SoI) toposheets, 1967 with a scale of 1:50,000 (Toposheet No. 58C/16); (2) LANDSAT 4–5 TM (1990), LANDSAT 7 ETM + (2004), and LANDSAT 8 OLI (2014) with a spatial resolution of 30 m. Rubber plantations within the Chandanapalli sub-watershed were introduced in 1962, and the earliest available topographic maps for this region after 1962 are for the year 1967. Hence, 1967 was taken as the base year for the analysis. The 1967 topographic map was surveyed in 1966–1967 and published by Surveyor General of India in 1967. SoI toposheets provide accurate topographic data, including information on LULC for all of India, and many studies used these toposheets to assess historical LULC changes (Hwan & Jo, 2015; Ghosh & Porchelvan, 2017; Pande et al., 2018). For the present study, SoI toposheets have been georeferenced, and areas under rubber plantations are demarcated using ArcGIS.

For the years 1990, 2004, and 2014, cloud-free LANDSAT-TM images with a spatial resolution of 30 m were downloaded from the Earth Explorer of USGS. The visible spectral bands of blue (0.45–0.52 μm), green (0.52–0.60 μm), and red (0.63–0.69 μm) were stacked in addition to the near-infrared (NIR) (0.77–0.90 μm) band with a 30-m spatial resolution, as different types of vegetation are more apparent in these bands (Horning, 2004). To avoid shifts between toposheets and satellite images, LANDSAT images were georeferenced using known points from the topographic maps. Both supervised classification and visual interpretation techniques were followed to delineate areas under rubber from satellite images. Due to the coarse resolution of LANDSAT images, spectral confusion of mixed pixels is very common while demarcating different vegetation types (Choodarathnakara et al., 2012). Hence, to avoid the issues of mixed pixels, visual interpretation was used in areas where confusion arose. False color composites (FCC) were created using green, red, and NIR bands for visual interpretation.

Furthermore, an extensive field investigation was conducted in the study area between 2012 and 2014 to gain field knowledge about the land use classes, and during household surveys, about 40 GPS locations were collected for areas with rubber plantations. These samples helped to cross-check the accuracy of the classification. Moreover, high-resolution Google Earth images helped to cross-check and improve the overall accuracy of the classified images. After preparing maps for areas under rubber plantations, they were demarcated in 1967, 1990, and 2014.

#### Analyzing the major impacts of the expansion of the rubber plantation

The changes in the physical, chemical, and biological state of the environment determine the quality of ecosystems and human well-being. These effects can be both positive and negative. To understand the impact of rubber plantations, household surveys of a total of 40 households with a total population of 183 people were conducted between 2013 and 2015 by using semi-structured questionnaires, questions regarding landholding size, type and details of land use and land use changes, drivers of land use changes, major environmental issues faced by the respondents, changes in socio-economic situation, awareness about natural resource management measures, etc. To understand the socio-economic and environmental impacts of rubber plantations, questions about the major positive and negative changes observed by the respondents after starting rubber cultivation were also included in the questionnaire. For instance, Have they experienced any changes in relation to their total annual household income and expenditure? Have there been changes in terms of living standards? Have they experienced changes in their social status? What are the biggest environmental issues they face after they start growing rubber? etc.

#### Assessing the drivers of land use changes to rubber plantations based on farmers’ perceptions

Drivers are crucial in understanding and managing natural resources and environmental problems. Drivers are defined as underlying factors or forces that, together with actors’ decisions, lead to pressures seen as proximate influences on environmental change (UNEP, 2012). Agricultural conversion, population growth, economic development, and globalization can be considered relevant drivers that exert varying degrees of influence on both the process and outcomes of land cover change (Jabbour & Hunsberger, 2014). The land use conversions within the Chandanapalli basin are very crucial, and underlying factors or drivers are mostly human-induced, but the effects of these factors have been exacerbated by the consequences of social and economic circumstances and, to a certain extent, by environmental changes. Based on household surveys and field observations, a set of direct and indirect drivers was identified within the basin.

To understand the drivers of land use changes, the questions placed before the respondents were as follows: When have they planted the rubber? Why did they start rubber cultivation? Are they getting any institutional support? Do they wish to convert rubber to any other crops? etc. Furthermore, three focus group discussions (FGDs), each consisting of 8–12 people, were conducted among rubber cultivators to understand their motivation and also to identify the direct and indirect drivers to change the land use to rubber plantations. For the analysis, drivers were classified into direct and indirect drivers, and they were also categorized as socio-economic, environmental, institutional, and policy drivers. Furthermore, analysis was also carried out to find out whether there was any change in the pattern of drivers over a period of time. For instance, the drivers that made people convert their land use in the 1970s may not be the same that affected people who changed their land use in the 1990s or 2000s. Hence, it was also assessed if there were any changes in the drivers over the course of time.

## Results and discussion

### Overall land use changes in Chandanapalli sub-watershed

**Fig. 2:**
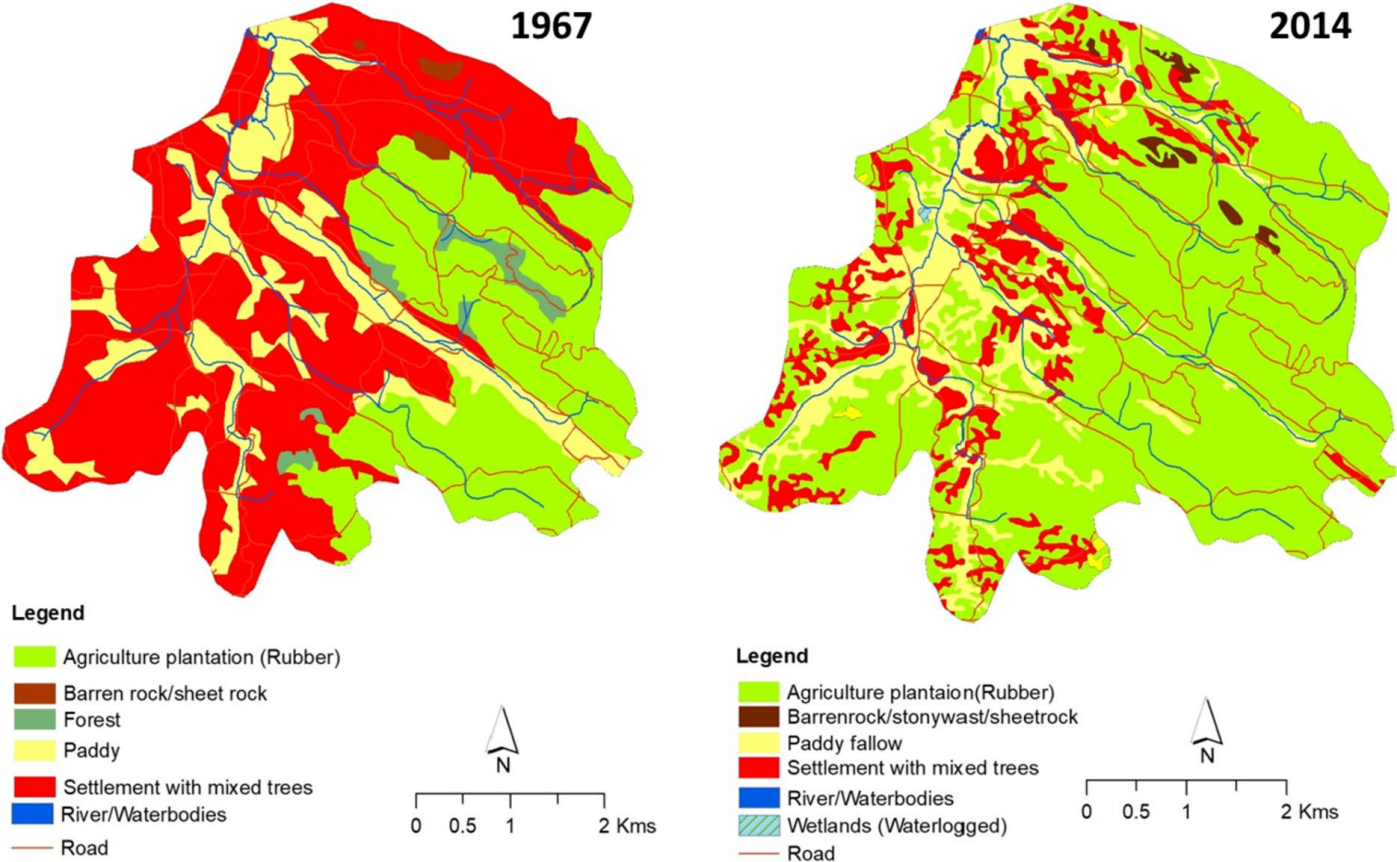
Land use changes within Chandanapalli between 1967 and 2014

We analyzed the overall LULC changes that occurred within the Chandanapalli sub-watershed over a period between 1967 and 2014. Analysis of land use change revealed that the watershed had experienced a significant change in LULC within 47 years. In 1967, more than 50% of the land area of this sub-watershed was covered with mixed trees, 30% was under rubber plantations, which were owned by PCKL, and 16.5% of the area was used for rice cultivation. It is also to be noted that 2.5% of the area was under dense evergreen forest. By 2014, more than 80% of the area under mixed tree areas had been converted to settlements with rubber plantations. Thus, the area under settlements with rubber plantations accounted for 74% in 2014, while only 11% of the area has remained under settlements with mixed trees. Similarly, paddy fields had been converted to cultivate seasonal crops such as tapioca, banana, pepper, and vegetables. In some areas within the sub-watershed, paddy fields were converted to coconut plantations. Figure 2 illustrates the LULC changes within the sub-watershed.

### Expansion of area under rubber plantations

**Fig. 3:**
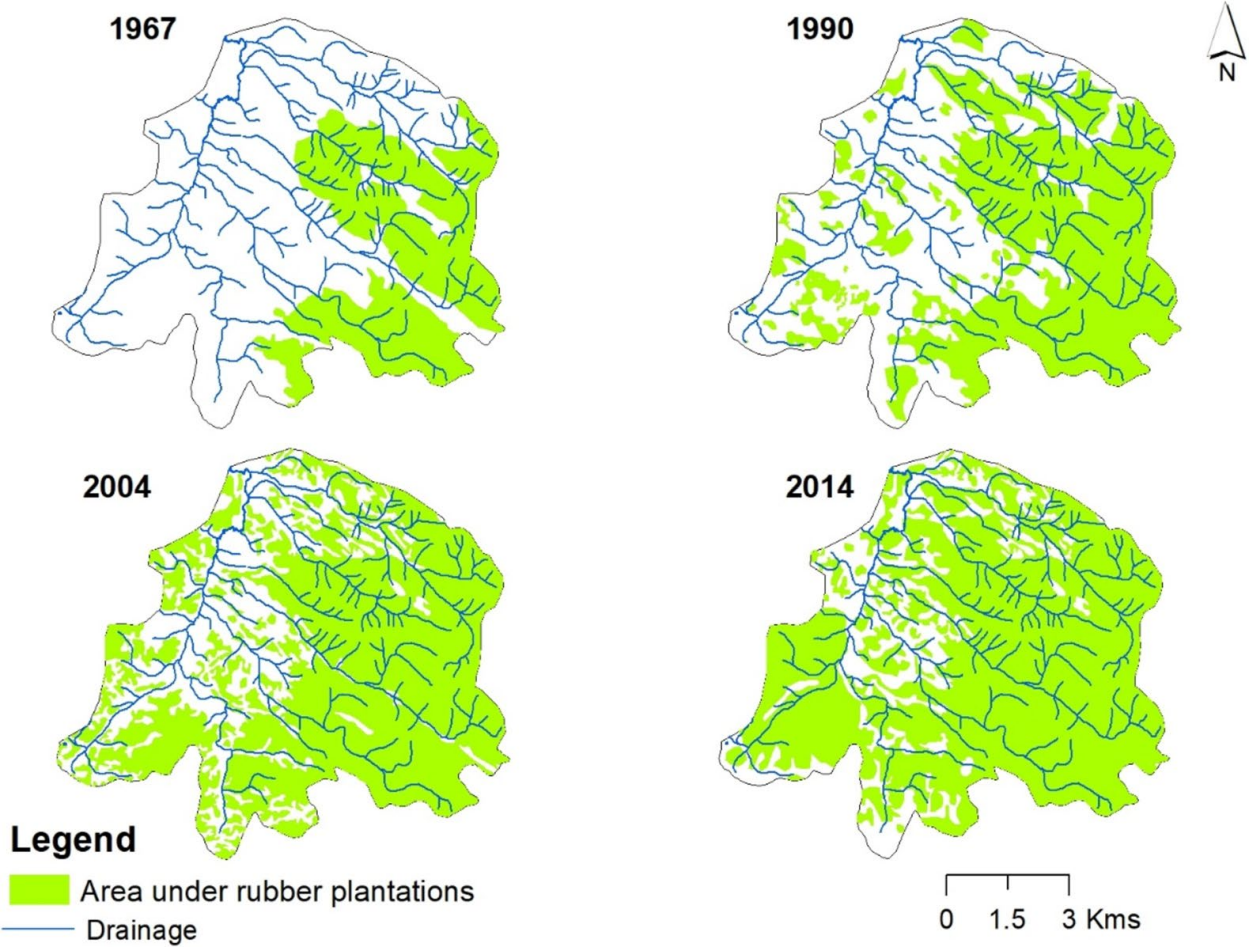
Temporal expansion of area under rubber plantations within Chandanapalli sub-watershed (1967–2014)

In 1962, the very first rubber plantation within the sub-watershed had a total area of 19 km^2^. After 1967, individual households began to plant rubber trees on their property. The results of the land use change analysis showed that rubber cultivation expanded to 35.5 km^2^ (54%) of the total watershed area in the 1990s and 52.2 km^2^ (74%) by 2014. This means that around 49% of private land holdings were used for rubber cultivation. Figure 3 shows the temporal expansion of rubber over the past five decades. The annual areal expansion rate of rubber between 1967 and 1990 was 1.1% of the total watershed area, which decreased slightly to 0.97% between 1990 and 2014. Over five decades, annually, approximately 0.94 km^2^ of land was converted to rubber plantation.

### Perceptions of farmers on the impacts of rubber plantation

#### Environmental impacts

**Fig. 4:**
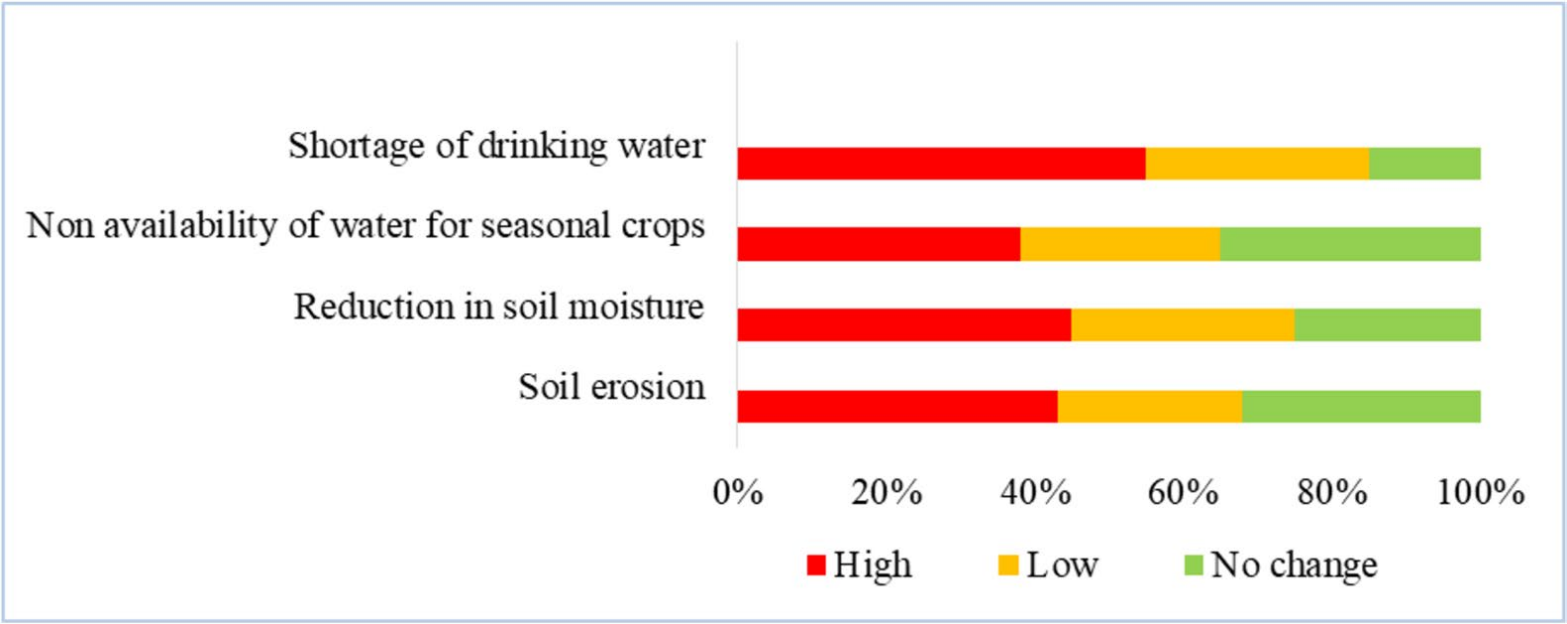
Environmental impacts observed by households

According to household surveys and FGDs, rubber plantations instigated various negative environmental impacts within the sub-watershed (Fig. 4). About 90% of households surveyed converted their traditional agricultural lands into rubber within the past two decades. Whereas only 10% of surveyed land holdings cultivate seasonal crops. Most of the farmers reported that they grow seasonal crops only in the initial phase of rubber replanting, and in later phases, excessive shade from rubber foliage restricts the cultivation of seasonal crops. Maps also show a significant expansion of rubber plantations throughout the study area (Fig. 3).

**Fig. 5:**
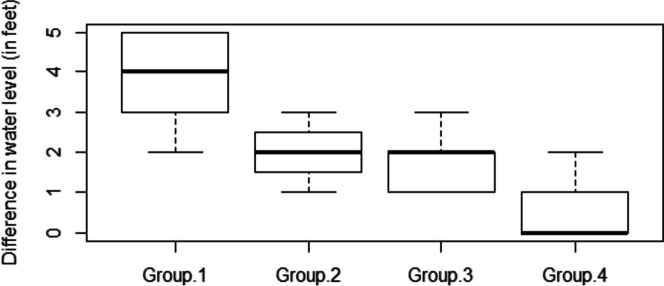
Boxplot showing the difference between well water levels between two time periods. Group 1: wells located in areas occupied by rubber plantations since before 1990; group 2: wells with areas under rubber plantations from 1990 to 2004; group 3: wells with areas under rubber plantations from 2004 to 2014; group 4: wells located within the areas of seasonal crops

Around 85% of respondents indicated that they were facing well water depletion, with 55% of respondents experiencing a serious shortage of drinking water and 30% of them experiencing a mild shortage of drinking water. From the household surveys and FGDs, it was observed that the water level decreased throughout the watershed between 1990 and 2014. Maximum well water depletion was observed toward the southeastern part of the watershed, while the minimum depletion was observed toward the western margin of the sub-watershed; 18% of households reported experiencing a drop in well water levels of up to 5 feet between 1990 and 2014, and these households were located near the margins of rubber plantations that were established during 1967; 25% and 27% of households experienced a drop of 3 feet and 2 feet respectively, and this was experienced by the households located in recently converted areas (2004–2014). Only 8% stated that they were not exposed to any water fluctuations during the period examined, and these households were located in areas with seasonal crops. It was significant to observe that some households near the river bank experienced a change in water level of up to 4 feet over the last 25 years. In addition, nearly 65% of surveyed households experienced severe water shortages from February to June, and 32% of households had deepened their wells due to water shortages over the past 10 to 15 years. According to elder respondents, areas converted to rubber plantations before the 1990s are subject to greater water stress than areas with more recent conversions. Boxplot (Fig. 5) illustrates that the higher mean difference in well water depletion is in the group-1 category, i.e., the areas with the longest duration under rubber plantations, and the lowest mean difference is for the group-4, i.e., the areas with seasonal crops.

Wells and ponds are the sources of irrigation for almost 90% of the households surveyed, and only 10% of the households rely on the river for irrigation. However, according to the respondents, the reduction in well water resulted in insufficient water availability to irrigate seasonal crops during the summer season; 38% of households reported that, due to severe water shortage for irrigation, they had to stop growing seasonal crops.

About 68% of the respondents also indicated that soil erosion increased during the rainy season, of which 43% of them claimed to be severely affected and 25% were slightly affected. People observe more soil loss in the plots where mixed trees are replaced with rubber, while people who switched their land use from seasonal crops such as tubers or bananas do not experience increased erosion. During the replanting of rubber, they observed that it was difficult to grow other crops on the same soil, and the productivity of other crops was found to be very low. Further reduction in soil moisture was also observed by the locals; 45% of respondents faced this problem intensively, and they reported that when they grow seasonal crops such as vegetables or bananas, they need to be watered more frequently than before.

#### Socio-economic impacts

**Fig. 6:**
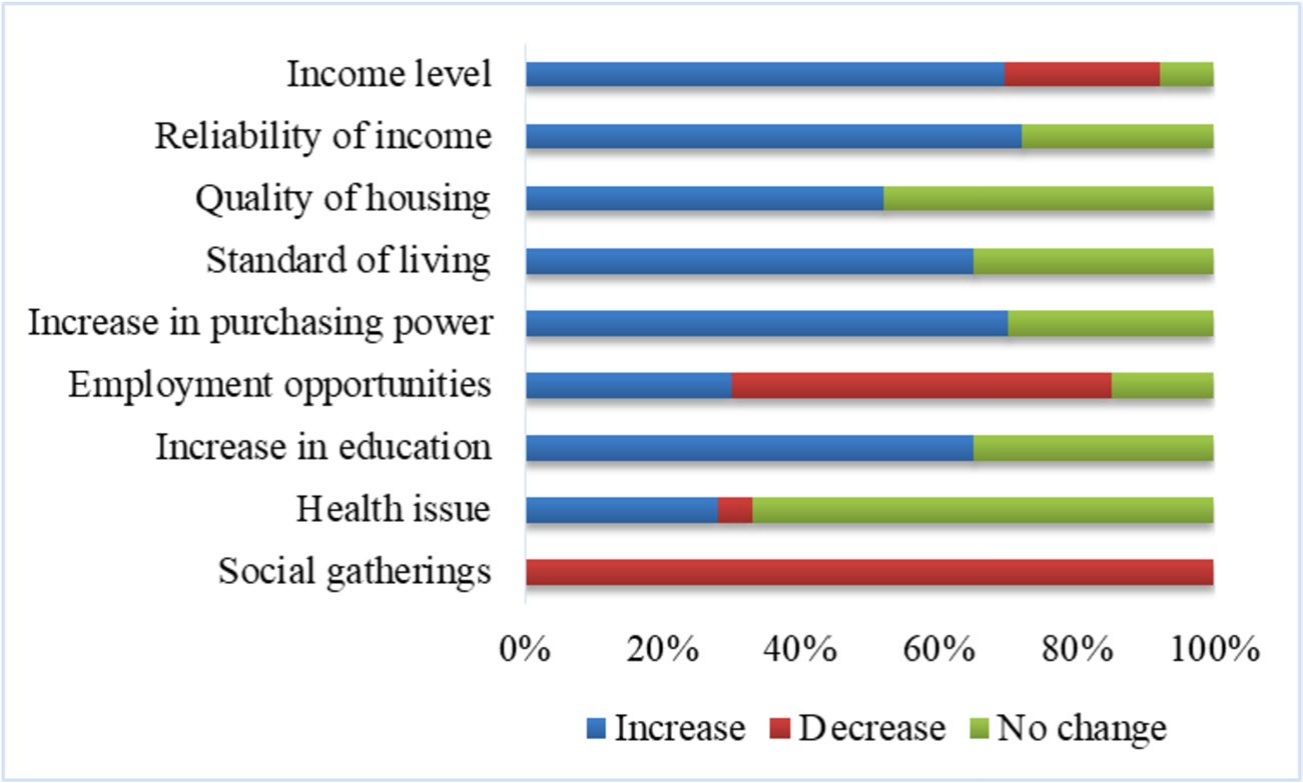
Perceived socio-economic impacts of rubber plantations according to households

From the household surveys and FGDs, it was observed that rubber plantations brought a big difference in the socio-economic conditions of the farmers, especially in terms of standard of living and economic growth, as illustrated in Fig. 6.

According to farmers, one of the notable positive effects of rubber cultivation was the increased income levels; 71% of respondents agreed that they could earn more income from rubber farming, and they have much more financial stability than before. During 2014–2015, while farmers earned 2,00,000 INR (3000 USD approx.) on average from rubber cultivation, their income from the seasonal crops was only less than 15,000 INR (230 USD). According to farmers, rubber is a perennial crop and less labor intensive. They tap latex only for 150 days per year. Hence, it gives people more opportunities for off-farm employment. It was observed that all the rubber cultivators depended on rubber cultivation as a secondary source of income. Of the rubber growers surveyed, about 37% rely on the service sector, 16% work as agricultural laborers, 15% rely on industrial and construction industries, and 12% work as rubber tappers as their primary source of income. However, 30% of respondents claimed that despite off-farm employment opportunities, there has been a decline in agriculture and allied jobs due to the reduction in seasonal crops. Overall, all respondents agreed that the quality of life had improved after rubber cultivation, albeit to varying degrees. According to respondents, rubber cultivation contributed to a higher and steady income, which allowed them to spend more money on children’s education and for home improvements, to buy home appliances and jewelry, etc. All respondents mentioned that the general level of education in the study region has increased. For example, among the total surveyed population, 50% of the population had a level of higher secondary or above. According to farmers, the additional income from rubber allows them to pay tuition fees for schools and colleges, thereby enabling their children to choose educational institutions and courses they like.

However, our results also show, despite the increase in income and standard of living, that rubber workers are subjected to several health issues. Notably, about 28% of respondents experienced respiratory problems. According to farmers, they collect the latex from the trees by tapping and then coagulate it by mixing it with formic acid which causes foul smells and leads to breathing difficulties. Furthermore, all respondents reported that there was a decrease in socialization and group activities after changing their land use. According to elderly farmers, when they grew seasonal crops such as bananas, tubers, or vegetables, they tended to interact more with neighbors and other farmers and share seeds and parts of the harvests. However, such interactions no longer exist, especially after rubber cultivation, which is a market-oriented product with little scope for sharing.

### Perceptions of farmers on drivers of land use changes within the sub-watershed

The first phase of land use conversion into rubber plantations within the study area was driven by government policy. In particular, in 1962, about 19 km^2^ of forest areas in the study area were cleared and planted with rubber trees. The history of rubber cultivation in India shows that as early as World War II, the British recognized the need and opportunities for natural rubber cultivation in India, particularly for strategic and security reasons, and the rubber growers were encouraged to produce as much rubber as needed for the use in the war. Faced with the demand for natural rubber, natural rubber growers managed to get good yields, and the Indian government recognized the importance of natural rubber in defense and modern industry. After the World War, the Rubber Board (RB) was founded by the Indian government in 1947 due to constant pressure from natural rubber growers for the establishment of a permanent organization to protect the interests of the industry. Since then, RB has been constantly striving to increase the natural rubber area and improve natural rubber production. Government policy was, therefore, the driver behind the start of rubber cultivation within the watershed. In our analysis, we considered only farmer perceptions to understand the underlying drivers for land use conversions by farmers.

Perceptions of farmers on drivers of large-scale land use conversions within the sub-watershed were collected based on FGDs among rubber cultivators and household surveys. During FGDs and household surveys, people were asked to point out the reasons why they changed the land use and why they prefer rubber over other crops? People’s responses were categorized into demographic, socio-economic, environmental, institutional, and policy drivers, as provided in Table 2.

**Table 2:** Farmers’ perception on major drivers of land use conversions within Chandanapalli sub-watershed watershed

Major drivers	Sub-drivers	Population claimed (%)
Demographic drivers	Shortage of labor for other cultivation	100
	Population growth and land fragmentation	76
Socio-economic	Higher profit from rubber	100
	Reduction in agricultural production	78
	Off-farm employment opportunities	72
	Increased standard of living and education	15
Institutional and policy drivers	Input subsidies for rubber cultivation (free seedlings, fertilizers, etc.)	92
	Good marketing system for latex	84
	Less support for other crops	55
Environmental drivers	Water shortage during the summer season	56
	Decline in soil fertility	48
	Soil erosion	30
	Forced to plant rubber as neighbors planted	23

#### Demographic drivers and socio-economic drivers

During FGDs, all participants cited the lack of cheap labor to grow seasonal crops as one of the major reasons for switching to rubber cultivation in the earlier phases; 76% of respondents identified population growth and land fragmentation as strong demographic factors influencing land use conversion. The average size of land holdings per household in the surveyed population ranged from 1 to 1.5 acres; 18% of households owned less than 50 cents (0.2 hectare) of land. According to the respondents, in contrast to rubber, seasonal crops such as rice or vegetables are only profitable if they are grown on larger farms. Smaller plots limit the use of machinery and are not profitable. Out of the total respondents, 22% stated that in the past, they cultivated rice, vegetables, and tubers in their fields during the off-season. However, when their fields were divided among their descendants, they were forced to give up rice cultivation and other seasonal crops as it was not profitable to cultivate paddy on fragmented land holdings.

Another very important and direct driver for land use conversion was the higher profits from rubber compared to other seasonal crops. All respondents who have been growing rubber for more than 10 years agreed that they could earn more income from growing rubber and that it was a much more stable income than before. Respondents also claimed that they experience a high level of uncertainty in income generation from seasonal crops due to crop failures, pest and disease attacks, climate variability, etc. Many seasonal agricultural products are perishable, so if farmers cannot sell them immediately, they can expect a loss. Furthermore, they could not store their produce until they got higher prices because of the lack of proper storage facilities. Whereas rubber is a perennial crop, less labor intensive, and more cost-efficient. In addition, latex is non-perishable and requires less space for storage, allowing farmers to wait to sell their products when the price is higher. Thus, according to the rubber cultivators, the higher profit from rubber was not just in terms of its sale value but also in terms of cost of production and flexibility in sales.

Off-farm employment opportunities associated with rubber plantations were found to be another attractiveness to choosing rubber over other crops for 72% of respondents. As most households depended on rubber as a secondary source of income, this additional income had a positive influence on people’s standard of living. With this additional income, these households could spend more on children’s education, food, maintaining a home, etc. This extra income helped to lift the economic status of these households. During FGDs, it also emerged that the increased standard of living of rubber cultivators and improved education were found to be an important indirect driver. As with the rest of Kerala, educated people tend to quit agriculture and search for other jobs. Thus, it became more convenient to plant perennial crops like rubber that require less time and labor; 15% of people claimed that the increased standard of living of rubber cultivators motivated them to quit seasonal crops and plant rubber trees.

#### Institutional and policy drivers

We learned from the respondents that the Rubber Board (RB) plays an important role in attracting people to plant rubber. All respondents were satisfied with the facilities provided by the RB. All rubber cultivators took part in FGD, and surveys were availed of strong support and subsidies from the RB. Support from the RB includes input subsidies like the distribution of high-yielding varieties of rubber seedlings, cash subsidies for fertilizers, and financial incentives and compensation for replanting rubber when tree failures occur due to natural disasters, which were attractive to growers. About 38% of the respondents experienced tree failures at different points in time, and all received compensation for tree failure. Good networking of RB with the rubber growers and the training programs offered by them all found to be appealing for the cultivators.

Similarly, a good marketing system provided by the RB for collecting latex was found to be another criterion for encouraging rubber cultivation. Rubber Board has stores in Chandanapalli to collect the rubber produces; 97% of the surveyed sold their products to collection centers of RB. The RB regulates prices during market failure by fixing a minimum price for the produce, and hence, the growers do not face much loss as in the case of other crops.

Farmers opined that they receive less support for other seasonal crops from *Krishi Bhavan* (Agriculture Office), and this is one of the reasons why they shifted to rubber cultivation. Even though 42% of respondents requested cash support during crop failures, only 18% of them availed, and none of them availed of the amount they had requested. It is found to be a major contributing driver in encouraging rubber cultivation. Moreover, they also claimed that support from the *Krishi Bhavan* was largely based on the economic ceiling of the household, and most of the time, people did not get subsidies or other benefits if they fell out of the ceiling. According to the participants, a few times, *Krishi Bhavan* collected soil samples from their properties, but they were never informed about the results and intimated how to improve their cultivation. Thus, all participants in surveys and FGD expressed their dissatisfaction with the services provided by *Krishi Bhavan* contrary to their satisfaction with the support of the RB.

#### Environmental drivers

Our results show that environmental drivers also played an important role in changing the land use within the study area. Nearly half of respondents experienced reduced soil fertility and moisture, resulting in up to a 40% decrease in yield for seasonal crops, specifically tubers and vegetables. The scarcity of water for irrigation of seasonal crops also prompted farmers to grow rubber, and about 14% of respondents converted their land use to rubber recently because rubber does not require irrigation. Notably, 23% of respondents were forced to convert their farmland to rubber due to the spreading of rubber in adjacent plots. Since rubber trees have an elaborate root system, they require more water than the seasonal crops, and in summer, excessive uptake of soil moisture by the rubber trees leaves the soil in the adjacent plot drier/extremely dry. This decline in soil moisture also prevents farmers from growing seasonal crops and opting for rubber.

### Changing nature of drivers

**Fig. 7:**
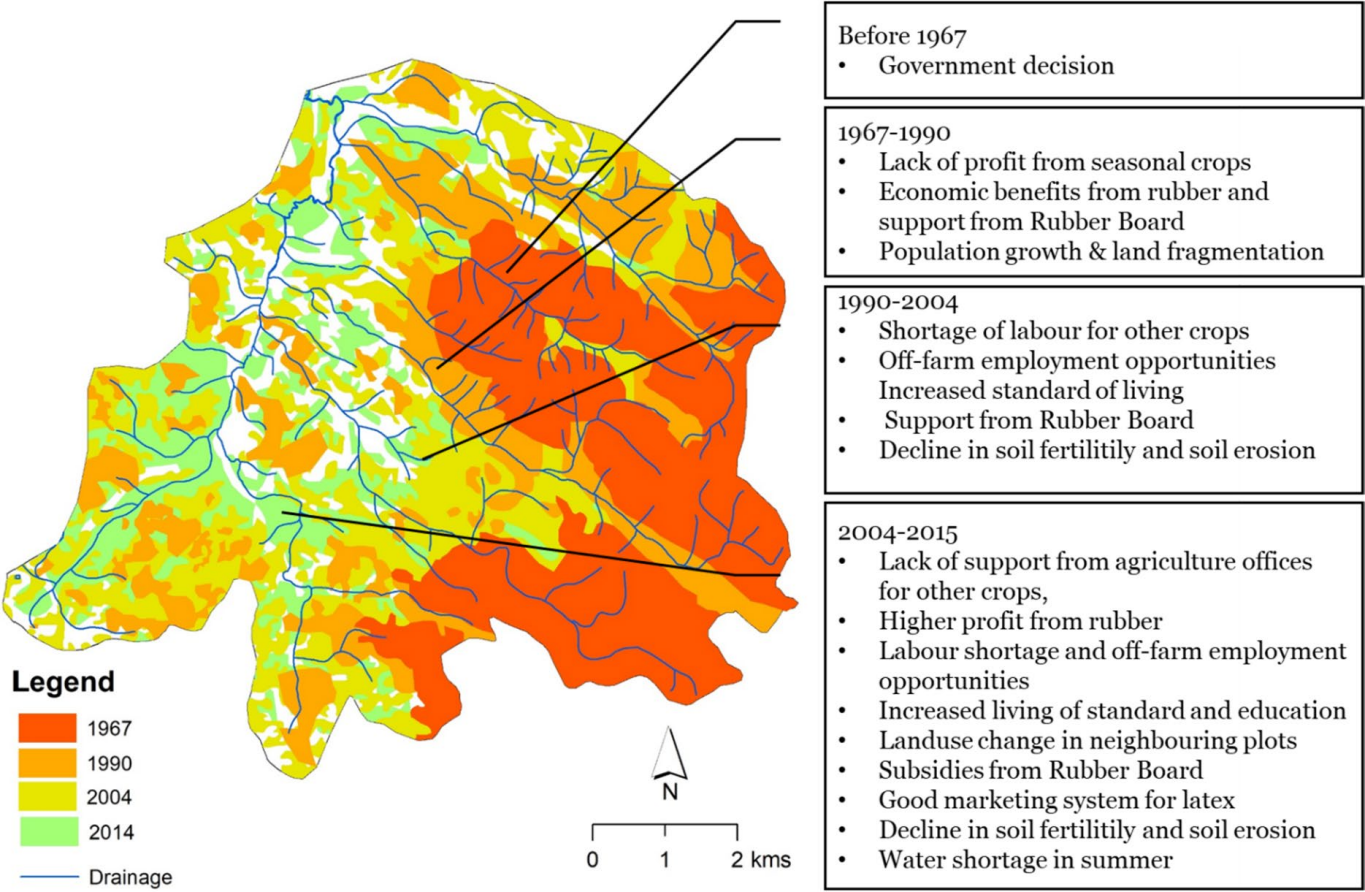
Drivers of land use changes in relation to the expansion of area under rubber plantations within Chandanapalli sub-watershed

The changing pattern of drivers over the past few decades within the watershed has been assessed and presented in Table 2 and Fig. 7. As can be seen from the maps, rubber expansion was gradual within the study area. Based on FGD, household surveys, and expert interviews, an assessment of the main trends in the drivers was made. This exercise was conducted based on disaggregated data on factors contributing to land use change over different time periods. More specifically, opinions from people who had converted land use in different time periods studied were collected and grouped to arrive at the results.

Drivers contributing to land use conversion between 1967 and 1990 were primarily associated with demographic and socio-economic drivers, including land fragmentation and the economic advantages of rubber compared to other crops. Whereas from 1990 to 2004, there was a driver change. For example, employment opportunities outside of agriculture, which rubber cultivation offers, as well as an increased standard of living, proved to be the most important drivers during this time. In addition, the role of institutions and the quality of the environment become visible as drivers at this time. Whereas for those who switched land use after 2004, environmental, institutional, and policy drivers have become more important in addition to demographic and socio-economic drivers. The role of institutions like RB and Krishi Bhavan has become increasingly important over the past decade. According to farmers, they did not experience scarcity of water for irrigation before 2004; thus, factors such as water scarcity for irrigation, cultivation on adjacent plots, etc. were only mentioned as drivers of land use conversion after 2004.

## Discussion

### Major impacts of rubber plantations

The major objectives of our study were to understand the impacts of the expansion of area under rubber plantations and to understand the drivers of land use conversions. Our analysis showed that the Chandanapalli sub-watershed experienced a higher rate of land use conversion to rubber plantations from seasonal crops and mixed trees within the last five decades. By 2014, about 74% of the sub-watershed was under rubber plantations. Even though there has been a slight decline in the rate of land use conversion since 1990, almost a third of the watershed is still under rubber. These results are very relevant as this is not only the case in the study area, but rapid land use conversion to rubber is also occurring in many midland regions of Kerala (Chattopadhyay, 2015).

Based on household surveys and FGD, our results showed that the expansion of rubber plantations has led to multiple environmental and socio-economic impacts. While rubber cultivation has helped to improve the socio-economic conditions of the farmers within the sub-watershed, it has also caused a large number of environmental problems. According to our results, rubber plantations have a positive impact on household income, stability of income flow, per capita expenditure, access to education, and the overall living standard of the farmers. Similar findings of other researchers have shown that rubber plantations lead to an increase in per capita income and expenditure (Liu et al., 2006). Conversely, our study also shows that despite the many socio-economic benefits of rubber plantations, it also led to reduced socialization among farmers. Traditionally, when farmers grew rice and other seasonal crops, they practiced group farming as it required more labor. From land preparation to harvesting, a large number of laborers and people were required to carry out these activities together as a collective operation. They also made joint decisions about the selection of rice varieties or other crops. Likewise, harvested products were shared among the neighbors, and all of this helped them maintain a high level of togetherness and a high level of information sharing about subsidies, seeds, or relevant schemes or programs. All of these also had a positive impact on their lives. However, rubber plantations require less labor and no group activities, and they offer fewer opportunities for socialization and dissemination of information.

Similarly, our results also show that rubber plantations lead to several environmental problems, including depletion of well water resources, reduction in soil moisture, and unavailability of water for seasonal crops. According to our study, almost 55% of the surveyed households that grow rubber suffer from a severe lack of drinking water during the dry season, especially from February to May. Among them, about 32% of families had deepened their wells in the past 10 to 15 years. According to our findings, areas occupied by rubber plantations over a longer period showed higher water scarcity and a reduction in soil moisture compared to areas occupied by recent plantations or seasonal crops. Rubber, as a deciduous tree, has a different phenological adaptation than evergreen trees and requires large amounts of water during leaf flushing, which coincides with the dry season in Kerala (Vinod et al., 1996; Williams et al., 2008). Root zone activities show significant deep root water uptake during wintering and flushing of rubber trees, and they conserve the amount of water withdrawn without being released into the atmosphere until new foliage has grown (Guardiola-Claramonte et al., 2010). In addition, rubber trees tend to take up deep water when soil moisture in the upper layer is insufficient, especially during the dry season (Wu et al., 2016), causing a depletion of water resources. Our findings also substantiate the results from a similar study conducted by the National Center for Earth Science Studies (Mahamaya & Sakunthala, 2014; Rajappan et al. 2012). They concluded that the rubber-dominated Chandanapalli sub-watershed has the lowest annual water yield compared to other watersheds that are under other land use types, such as natural forests, paddy fields, and other seasonal crops. However, these studies mainly considered the influence of land use and geomorphological factors on water yield. Studies have also shown that there is a significant decreasing trend of rainfall in most of the regions of Kerala, especially in the month of January, July, and November (Nair et al., 2014). However, due to a lack of rainfall data for the study area, in our study, we only mapped spatial variations of decline in water level and analyzed these in relation to the distribution of rubber plantations. This analysis showed that there is a spatial relationship between the age of rubber plantations and water levels. Further studies are needed to understand the overall impacts of climate change on surface and groundwater resources. Similarly, in-depth studies within the study area compared to other watersheds, incorporating other factors such as population growth and changes in domestic and industrial water use, are needed to determine the correlation between the expansion of rubber plantations and water depletion.

Furthermore, our study shows that the expansion of rubber plantations leads to changes in cultivation patterns, loss of biodiversity, and the emergence of monocultures. Studies have shown that the proliferation of rubber monocultures on formerly agro-diverse land reduces biodiversity and biological buffering against pests, weeds, and diseases (Aratrakorn et al., 2006). Although our study highlights that rubber plantations have brought farmers a positive economic situation, their monoculture nature may reduce farmers’ ability to cope with economic and climatic shocks (Qiu, 2009; Hoang et al., 2010; Fox, 2014). Our results also show that farmers face issues of soil degradation and increased soil erosion. A study by Nguyen et al. (2020) has shown that the effects of rubber plantations on soil health appear to be greatest in the first five years after the plantation is established. In addition, the cultivation of rubber plantations raises serious concerns about soil health and conservation, especially when converting natural forests to rubber plantations (Nguyen et al., 2020). Similarly, farmers in our study area faced severe soil erosion during the monsoon season in the areas where they converted mixed trees to rubber plantations due to the different root systems, suggesting that converting mixed trees to rubber could also lead to increasing soil erosion.

In our study, we observed respiratory problems among 28% of respondents caused by the chemicals (formic acid) and smoke during the processing of latex. These findings correspond to prior studies (Nair et al., 2016 & Gopalakrishnan et al., 2019) where chemical exposure was reported to be the major health hazard of rubber plantation workers. Notably, earlier studies also have shown similar results of a significantly higher prevalence of all chronic respiratory symptoms (Zuskin et al., 1994) among rubber workers.

### Changing nature of drivers

Drivers of land use change, including the role of the global market and international trade, economic viability, and socio-economic and political factors, were a focus of study by many authors (Lambin et al., 2003; Viswanathan & Bhowmik, 2014; Zhao et al., 2018). However, the periodical changes in drivers that influenced the expansion of rubber plantations have not been looked at by the authors. Our findings show that even though the land use conversions to rubber plantations continue, the drivers of land use conversions are changing over time within the study area. These changes in drivers can be broadly categorized into different groups, as listed below.

#### Demographic and socio-economic drivers

Our results showed that demographic and socio-economic drivers played an important role in the expansion of rubber plantations throughout the study period. There are several underlying reasons behind these socio-economic developments.

##### Economic benefits

One of the most influential drivers of land use conversion was the higher economic benefits of rubber plantations. Since the 1980s, food shortage was no longer an issue in Kerala, but low income was a major concern. Seasonal crops yielded less income due to higher operational costs in comparison to rubber, and crop failures were very common for seasonal crops due to extreme weather events or due to attacks from pests and rodents. Hence, since the late 1970s, private land owners started planting rubber trees within their plots motivated by higher profits from rubber plantations compared to other crops. It was also observed that economic benefits and lower labor requirements of rubber compared to other seasonal crops (Viswanathan & Bhowmik, 2014) remained to be a constant driver throughout the studied period.

##### Land reforms and fragmentation

The land reform movement was a milestone where all people including the landless people got ownership of landholdings. The first land reform act in the state began in 1963 and was amended in 1969. The act protected tenants from predatory rents for land leasing and also established a size ceiling for the possession of landholdings by individual families to avoid concentration of land in few hands. Thus, lands were redistributed among the landless which later got divided and subdivided among descendants due to inheritance law. However, when the land becomes small and fragmented, it is difficult to cultivate crops on a commercial scale. By the late 1980s, 74% of the state’s landholdings were divided into small plots of less than 2 ha. Within the sub-watershed, the average size of landholdings per household among the surveyed population was less than one hectare. Homestead cultivation in such small landholdings scarcely offered a means of livelihood, and farm outputs declined. It was interesting to note that 18% of the households had landholdings less than 0.25 ha. About 22% of total respondents were used to cultivating paddy in their fields while vegetables and tubers during off-season. Whereas, when their properties were divided among their descendants, they had to give up paddy farming as it was not profitable to cultivate paddy in fragmented landholdings.

##### “Gulf Boom” and shortage of agricultural laborers

During the mid-1970s and 1980s, people largely started to migrate from Kerala to Gulf countries in search of jobs, which is also referred to as a period of the “Kerala Gulf Boom.” This migration largely consisted of middle-class working groups and thus resulted in a shortage of agricultural laborers for labor-intensive crops. During FGDs, all participants pointed out the unavailability of cheap laborers for cultivating seasonal crops as one of the major reasons for changing to rubber.

##### Education and standard of living

Another major socio-economic factor that strongly influenced the agriculture sector was the increase in the literacy rate and improved standard of living. Even though Kerala had higher literacy rates historically since the 1970s, there were strong educational campaigns and grassroots movements like the 1990 literacy programs which helped to achieve a 100% literacy rate for the state. The impacts of these movements were visible within the studied area as well. According to the farmers, a higher rate of education negatively affected the agriculture sector in the sub-watershed, like the rest of the state. People have largely opted for jobs outside the primary sector which resulted not only in the shortage of labor but also in the preference of perennial crops. Thus, off-farm employment opportunities associated with rubber plantations were found to be a major attraction for 72% of respondents to choose rubber over other crops. It was observed that the rubber cultivators depend on rubber as a secondary source of income.

#### Institutional drivers

Land use conversion before the 1990s was primarily determined by demographic and socio-economic factors, while the role of institutions as well as environmental changes became more prominent and started to influence land use conversions after the 1990s. Notably, the key role played by the RB has been found to be a strong driver in recent years. A study by Viswanathan and Bhowmik (2014) in north-eastern India confirms that the sustained efforts of the RB seem to have enabled the rubber producers to achieve higher production and productivity. They also state that the success of rubber cultivation also emerged from the strong networking of RB with the growers.

#### Environmental drivers

Our results showed that after the 1990s, people started to observe a reduction in soil fertility and soil erosion as one of the main reasons for not choosing seasonal crops. Whereas, after 2004, farmers were more concerned about water depletion and reduction in soil moisture. Hence, our study shows that environmental drivers have started to play a major role in determining the land use within a particular area. Furthermore, during FGDs, it was observed that, even though participants were concerned about the well water depletion, none of the participants wanted to cultivate other crops or attempt intercropping methods. It was also found that, on the one hand, rubber plantations lead to the depletion of water resources, resulting in a lack of water for irrigation of other crops, but on the other hand, rubber does not require irrigation. This entices farmers to grow more rubber, and it creates a vicious circle. About 40% of participants who have been in farming for more than 30 years have even replanted rubber. They claimed that they had cultivated seasonal crops during the replanting of rubber trees, which is limited to the first three years from the planting of rubber saplings. After three years, they stopped cultivating other crops due to the foliage of rubber plants shading the ground, allowing little sunlight to support other crops. Wu et al. (2016) stated that intercropping legume plants with rubber trees can benefit rubber trees’ higher nitrogen supply, increase their water use efficiency, and better utilize soil water of each soil layer.

## Conclusions

This study analyzed the expansion of rubber plantations, its major impacts, and drivers for land use conversion to rubber plantations based on tropical sub-watersheds from southern India. According to our study, although rubber cultivation has contributed significantly to increasing the economic status and per capita income of the population, it has adversely affected the natural environment, biodiversity, and water resources within the study area. Our analysis showed that, in addition to economic incentives, institutional interventions also played a great role in promoting the expansion of rubber plantations. Although the economic benefits of rubber remained a constant factor throughout the study period, the institutional, policy and environmental factors became more visible over time. Therefore, further studies are needed to identify measures to promote the cultivation of other crops.

In order to adopt rational land use management, it is very important to understand the drivers of land use change. However, our study highlights that it is also important to consider the changing nature of the drivers for better land use management. It can also be noted that drivers for planting rubber plantations were different at different times and were composed of the decisions of several individual landowners. Hence, to make an intervention, it is important to understand the changing nature of drivers. Equally, it can also be stated that the lack of strong institutional support for other crops has also contributed to the decline in their area. In addition, it was also observed that rubber growers were reluctant to practice intercropping or plant other crops. Therefore, it is important to take collective actions by farmers and agricultural institutions to ensure sustainable agricultural practices by improving marketing opportunities for seasonal crops and creating income-enhancing opportunities for farmers.

In the past, social and economic growth has been at the forefront of many interventions, and environmental sustainability has not been considered. Our results showed that rubber plantations contributed to the increase in several environmental issues, including changes in water levels, alteration of cropping patterns, and reductions in soil moisture. Therefore, we conclude that government interventions to encourage additional rubber cultivation should be structured through government policies and regulations to ensure and minimize the risk of environmental issues that would significantly affect the economic prosperity of the region.

## Data Availability

The datasets generated during and/or analyzed during the current study are available from the corresponding author upon reasonable request.

## References

[CR1] Alijani Z, Hosseinali F, Biswas A (2020). Spatio-temporal evolution of agricultural land use change drivers: A case study from Chalous region, Iran. Journal of Environmental Management.

[CR2] Antje A, Hollingsworth PM, Ziegler AD, Fox JM, Chen H, Su Y, Xu J (2015). Current trends of rubber plantation expansion may threaten biodiversity and livelihoods. Global Environmental Change.

[CR3] Aratrakorn S, Thunhikorn S, Donald PF (2006). Changes in bird communities following conversion of lowland forest to oil palm and rubber plantations in Thailand. Bird Conservation International..

[CR4] Arowolo AO, Deng X (2018). Land use/land cover change and statistical modelling of cultivated land change drivers in Nigeria. Regional Environmental Change.

[CR5] Ayutthaya SIN, Do FC, Pannangpetch K, Junjittakarn J, Maeght JL, Rocheteau A, Cochard H (2011). Water loss regulation in mature Hevea brasiliensis: Effects of intermittent drought in the rainy season and hydraulic regulation. Tree Physiology.

[CR6] Beilin R, Lindborg R, Stenseke M, Pereira HM, Llausàs A, Slätmo E, Cerqueira Y, Navarro L, Rodrigues P, Reichelt N (2014). Analysing how drivers of agricultural land abandonment affect biodiversity and cultural landscapes using case studies from Scandinavia, Iberia and Oceania. Land Use Policy.

[CR7] Benini L, Bandini V, Marazza D, Contin A (2010). Assessment of land use changes through an indicator-based approach : A case study from the lamone river basin in Northern Italy. Ecological Indicators.

[CR8] Celine G, James EJ (2015). Assessing the implications of extension of rubber plantation on the hydrology of humid sub-tropical river basin. International Journal of Environmental Research.

[CR9] Chattopadhyay S (2015). Environmental consequences of rubber plantations in Kerala, NRPPD Discussion Paper 44.

[CR10] Choodarathnakara AL, Kumar TA, Koliwad S, Patil CG (2012). Mixed Pixels : A challenge in remote sensing data classification for improving performance. International Journal of Advanced Research in Computer Engineering & Technology.

[CR11] Delgado-Aguilar MJ, Hinojosa L, Schmitt CB (2019). Combining remote sensing techniques and participatory mapping to understand the relations between forest degradation and ecosystems services in a tropical rainforest. Applied Geography.

[CR12] Dhanya V, Renoy G (2015). Drainage development in Achankovil Shear Zone, South India. Current Science.

[CR14] Foley JA (2005). Global consequences of land use. Science.

[CR15] Fox JM (2014). Rubber plantations expand in mountainous southeast Asia: What are the consequences for the environment?.

[CR16] Fox TA, Rhemtulla JM, Ramankutty N, Lesk C, Coyle T, Kunhamu TK (2017). Agricultural land-use change in Kerala, India: Perspectives from above and below the canopy. Agriculture Ecosystem and Environment.

[CR17] Ghosh J, Porchelvan P (2017). Remote sensing and GIS technique enable to assess and predict landuse changes in Vellore district, Tamil Nadu, India. International Journal of Applied Engineering Research.

[CR18] Gopalakrisnan S, Kurien A, Paul D (2019). A study of pulmonary function test in workers engaged in processing of natural rubber. International Journal of Physiology.

[CR19] Guardiola-claramonte M, Troch PA, Ziegler AD, Giambelluca TW, Durcik M, Vogler JB, Nullet MA (2010). Hydrologic effects of the expansion of rubber (Hevea brasiliensis ) in a tropical catchment. Ecohydrology.

[CR20] Guardiola-Claramonte M, Troch PA, Ziegler AD, Giambelluca TW, Vogler JB, Nullet MA (2008). Local hydrologic effects of introducing non-native vegetation in a tropical catchment. Ecohydrology.

[CR21] Gunnell Y, Ignacio JAF, Delbart N, Ogania JL, Henry S (2017). Monitoring land-use change by combining participatory land-use maps with standard remote sensing techniques: Showcase from a remote forest catchment on Mindanao Philippines. International Journal of Applied Earth Observation and Geoinformation.

[CR22] Häuser I (2015). Environmental and socio-economic impacts of rubber cultivation in the Mekong region: Challenges for sustainable land use. CAB Rev.

[CR23] Hoang MH, Do TH, van Noordwijk M, Pham MT, Palm M, To XP, Doan D, Nguyen Thanh TX, Hoang TVA (2010). An assessment of opportunities for reducing emission from all land uses – Vietnam preparing for REDD. Final National Report.

[CR24] Horning, N. (2004). Selecting the appropriate band combination for an RGB image using Landsat imagery Version 1.0. American Museum of Natural History, Center for Biodiversity and Conservation.

[CR25] Hwan SV, Jo E (2015). Survey of land use and land cover change detection using remote sensing. SSRG International Journal of Geoinformatics and Geological Scence.

[CR26] Jabbour J, Hunsberger C (2014). Visualizing relationships between drivers of environmental change and pressures on land-based ecosystems. Natural Resources.

[CR27] Karunakaran N (2017). Trend, determinants and price-volatility of rubber-crop in Kerala. Journal of Economic and Social Development.

[CR28] Lambin EF, Geist HJ, Lepers L (2003). Dynamics of land-use and land-cover change in tropical regions. Annual Review Environmental Resources.

[CR29] Li, X., Wang, Y., Li, J., & Lei, B. (2016). Physical and socioeconomic driving forces of land-use and land-cover changes: A case study of Wuhan City, China. *Discrete Dynamics in Nature and Society,* 1–11. 10.1155/2016/8061069

[CR30] Liu, W., Li, J., Lu, H., Wang, P., Luo, Q., Liu, W.,& Li, H. (2013). Vertical patterns of soil water acquisition by non-native rubber trees (Hevea brasiliensis) in Xishuangbanna, Southwest China. *Ecohydrology,**7*(4). 10.1002/eco.1456

[CR31] Liu X, Jiang L, Feng Z, Li P (2016). Rubber plantation expansion related land use change along the Laos-China Border Region. Sustainability.

[CR32] Liu W, Hu H, Ma Y, Li H (2006). Environmental and socioeconomic impacts of increasing rubber plantations in Menglun Township, Southwest China. Mountain Research and Development.

[CR33] Ma X, Lacombe G, Harrison R, Xu J (2019). Expanding rubber plantations in Southern China : Evidence for hydrological impacts. Water.

[CR34] Malek Z, Boerboom L (2015). Participatory scenario development to address potential impacts of land use change: An example from the Italian Alps. Mountain Research and Development.

[CR35] Munthali MG, Davis N, Adeola AM, Botai JO, Kamwi JM, Chisale HLW, Orimoogunje OOI (2019). Local perception of drivers of land-use and land-cover change dynamics across Dedza district Central Malawi Region. Sustainability.

[CR36] Nair, T.S., Garg, S., Singh, M. M. (2016). A study of the health profile of rubber plantation workers in rural Kerala. *Asian Journal of Medical Sciences*, *7*, 3. 10.3126/ajms.v7i3.13288

[CR37] Nair A, Joseph KA, Nair KS (2014). Spatio-temporal analysis of rainfall trends over a maritime state (Kerala) of India during the last 100 years. Atmospheric Environment.

[CR38] Mahamaya C, Sakunthala C (2014). Valley formation and geomorphic processes under tropical wet and dry climate: Example from Kerala, Technical Report PLAN-283.

[CR39] Nguyen TT, Do TT, Harper R, Pham TT, Linh TVK, Le TS, Thanh LB, Giap NXS (2020). Soil health impacts of rubber farming: The implication of conversion of degraded natural forests into monoculture plantations. Agriculture.

[CR40] Pande CB, Moharir KN, Sanjay SFRK (2018). Study of land use classification in an arid region using multispectral satellite images. Applied Water Scence.

[CR41] Qiu J (2009). Where the rubber meets the garden. Nature.

[CR42] Rajappan, S., Nair, I. P., & Raju, K. (2012). Influence of watershed characteristics on annual discharge of Achankovil river basin, Kerala. In S. Chattopadhyay, & M. Chattopadhyay (Eds.), *Proceedings of 34th Institute of Indian Geographers Meet (2012)*, Centre for Earth Science Studies, Trivandrum, pp. 59.

[CR43] Sayer J, Nelson PN, Sayer J, Ghazoul J, Nelson P, Klintuni A (2012). Oil palm expansion transforms tropical landscapes and livelihoods Oil palm expansion transforms tropical landscapes and livelihoods. Global Food Security.

[CR44] Song XP, Hansen MC, Stehman SV (2018). Global land change from 1982 to 2016. Nature.

[CR45] Tan Z, Zhang Y, Song Q, Liu W, Deng X, Tang J, Deng Y, Zhou W, Yang L, Yu G (2011). Rubber plantations act as water pumps in tropical China. Geophysical Research Letters.

[CR46] Turner BL, Lambin EF, Reenberg A (2007). The emergence of land change science for global environmental change and sustainability. Proceedings of the National Academy of Sciences USA.

[CR47] UNEP, Fifth Global Environment Outlook Report. (2012). Global Environment Outlook 5 (GEO 5): Environment for the Future We Want. https://wedocs.unep.org/20.500.11822/8021

[CR48] Vijayan D, Kaechele H, Girindran R, Chattopadhyay S, Lukas MC, Arshad M (2021). Tropical forest conversion and its impact on indigenous communities Mapping forest loss and shrinking gathering grounds in the Western Ghats, India. Land Use Policy.

[CR49] Vinod KK, Meenattoor JR, Pothen J, Krishnakumar AK, Sethuraj MR (1996). Performance analysis for wintering pettern in Hevea Brasiliensis clones. Indian Journal of Natural Rubber.

[CR50] Viswanathan, P. K., & Bhowmik, I. (2014). Compatibility of institutional architecture for rubber plantation development in North East India from A Comparative Perspective of Kerala. *NRPPD Discussion Paper 38, Centre for Development Studies* (p. 71). Trivandrum: Centre for Development Studies.

[CR51] Wang Y, Hu Y, Niu X, Yan H, Zhen L (2022). Land use/cover change and its driving mechanism in Thailand from 2000 to 2020. Land.

[CR52] Williams LJ, Bunyavejshewing S, Baker PJ (2008). Deciduousness in a seasonal tropical forest in western Thailand interannual and intraspecific variation in timing, duration and environmental cues. Oecologia.

[CR53] Wu J, Liu W, Chen C (2016). Below-ground interspecific competition for water in a rubber agroforestry system may enhance water utilization in plants. Scientific Reports.

[CR54] Zaehringer JG, Llopis JC, Latthachack P, Thein TT, Heinimann A (2018). A novel participatory and remote-sensing-based approach to mapping annual land use change on forest frontiers in Laos, Myanmar, and Madagascar. Journal of Land Use Science..

[CR55] Zhao X, Pu J, Wang X, Chen J, Yang LE, Gu Z (2018). Land-use spatio-temporal change and its driving factors in an artificial forest area in Southwest China. Sustainability.

[CR56] Zhao Y, Han Z, Xu Y (2022). Impact of land use/cover change on ecosystem service value in Guangxi. Sustainability.

[CR58] Zuskin E, Mustajbegovic J, Jelinic DJ (1994). Respiratory function in rubber processing workers. Lijecnicki Vjesnik.

